# Supplementary material for: SyNDI: synchronous network data integration framework
Source: BMC Bioinformatics. 2018 Nov 6;19:403. doi: 10.1186/s12859-018-2426-5 (PMC6219086; doi:10.1186/s12859-018-2426-5)
Supplement: Supplementary file 5 — Files of the example presented in Additional file 4. This additional file is a zip package that contains all files used in Additional file 4. Detailed descriptions of each of these files are presented in a README file included in this zip package. (GZ 30411 kb) [file 12859_2018_2426_MOESM5_ESM.gz › SyNDITools/Meme2Fimo/meme_4.9.0_4.tar.gz!/meme_4.9.0/doc/examples/glam2_example_output_files.tgz!/glam2_example_output_files/glam2.html]

GLAM2


# GLAM2: Gapped Local Alignment of Motifs

Version 1056
  
 bin/glam2 -Q -O . -M -A 'meme@nbcr.net' -X '' -z 2 -a 2 -b 50 -w 20 -r 10 -n 2000 -D 0.1 -E 2 -I 0.02 -J 1 p sequences

If you use this program in your research, please cite: **MC Frith, NFW Saunders, B Kobe, TL Bailey, "Discovering sequence motifs with arbitrary insertions and deletions", PLoS Computational Biology, **4**(5):e1000071, 2008.**

## Best Motif Found:

| NAME | START | SITES | END | STRAND | MARGINAL SCORE |  |  |  |  |  |  |  |  |  |  |  |  |  |  |  |  |  |  |  |  |  |  |  |  |  |  |  |  |  |  |  |  |  |  |  |  |  |  |  |  |  |  |  |  |  |  |  |  |  |  |  |  |  |  |  |  |  |  |  |  |  |  |
| --- | --- | --- | --- | --- | --- | --- | --- | --- | --- | --- | --- | --- | --- | --- | --- | --- | --- | --- | --- | --- | --- | --- | --- | --- | --- | --- | --- | --- | --- | --- | --- | --- | --- | --- | --- | --- | --- | --- | --- | --- | --- | --- | --- | --- | --- | --- | --- | --- | --- | --- | --- | --- | --- | --- | --- | --- | --- | --- | --- | --- | --- | --- | --- | --- | --- | --- | --- |
|  |  |  |  |  |  |  |  |  |  |  |  |  |  |  |  |  |  |  |  |  |  |  |  |  |  |  |  |  |  |  |  |  |  |  |  |  |  |  |  |  |  |  |  |  |  |  |  |  |  |  |  |  |  |  |  |  |  |  |  |  |  |  |  |  |  |  |  |
| --- | --- | --- | --- | --- | --- | --- | --- | --- | --- | --- | --- | --- | --- | --- | --- | --- | --- | --- | --- | --- | --- | --- | --- | --- | --- | --- | --- | --- | --- | --- | --- | --- | --- | --- | --- | --- | --- | --- | --- | --- | --- | --- | --- | --- | --- | --- | --- | --- | --- | --- | --- | --- | --- | --- | --- | --- | --- | --- | --- | --- | --- | --- | --- | --- | --- | --- | --- |
| At1g01140.1\_ | 114 | L | K | E | D | E | A | R | R | Y | F | Q | Q | L | I | N | A | V | D | Y | C | H | . | . | . | S | R | G | V | Y | H | R | D | L | K | P | E | N | L | I | L | D | A | N | G | . | . | . | . | . | . | . | . | V | L | K | V | S | D | F | G | L | S | A | 165 | + | 118. |
| At1g01140.2\_ | 114 | L | K | E | D | E | A | R | R | Y | F | Q | Q | L | I | N | A | V | D | Y | C | H | . | . | . | S | R | G | V | Y | H | R | D | L | K | P | E | N | L | I | L | D | A | N | G | . | . | . | . | . | . | . | . | V | L | K | V | S | D | F | G | L | S | A | 165 | + | 118. |
| At1g01140.3\_ | 114 | L | K | E | D | E | A | R | R | Y | F | Q | Q | L | I | N | A | V | D | Y | C | H | . | . | . | S | R | G | V | Y | H | R | D | L | K | P | E | N | L | I | L | D | A | N | G | . | . | . | . | . | . | . | . | V | L | K | V | S | D | F | G | L | S | A | 165 | + | 118. |
| At1g01450.1\_ | 121 | L | S | L | P | V | A | V | D | L | M | L | Q | I | A | L | G | M | E | Y | L | H | . | . | . | S | K | R | I | Y | H | G | E | L | N | P | S | N | I | L | V | K | P | R | S | N | Q | S | G | D | G | Y | L | L | G | K | I | F | G | F | G | L | N | S | 180 | + | 41.2 |
| At1g01540.1\_ | 249 | L | T | W | D | I | R | M | N | I | I | L | G | M | A | K | G | L | A | Y | L | H | E | G | L | E | P | K | V | V | H | R | D | I | K | S | S | N | I | L | L | D | R | Q | W | . | . | . | . | . | . | . | . | N | A | K | V | S | D | F | G | L | A | K | 303 | + | 105. |
| At1g01540.2\_ | 249 | L | T | W | D | I | R | M | N | I | I | L | G | M | A | K | G | L | A | Y | L | H | E | G | L | E | P | K | V | V | H | R | D | I | K | S | S | N | I | L | L | D | R | Q | W | . | . | . | . | . | . | . | . | N | A | K | V | S | D | F | G | L | A | K | 303 | + | 105. |
| At1g01560.1\_ | 138 | L | T | D | D | H | S | R | F | F | L | Y | Q | L | L | R | G | L | K | Y | V | H | . | . | . | S | A | N | V | L | H | R | D | L | K | P | S | N | L | L | L | N | A | N | C | . | . | . | . | . | . | . | . | D | L | K | I | G | D | F | G | L | A | R | 189 | + | 82.7 |
| At1g01740.1\_ | 149 | M | K | W | A | M | R | L | R | V | A | L | Y | I | S | E | A | L | E | Y | C | S | N | N | . | G | H | T | L | Y | H | . | D | L | N | A | Y | R | V | L | F | D | E | E | C | . | . | . | . | . | . | . | . | N | P | R | L | S | T | F | G | L | M | K | 201 | + | 9.84 |
| At1g02970.1\_ | 344 | V | S | E | R | E | I | L | V | I | M | H | Q | I | A | K | A | L | H | F | V | H | . | . | . | E | K | G | I | A | H | L | D | V | K | P | D | N | I | Y | I | K | N | G | . | . | . | . | . | . | . | . | . | V | C | K | L | G | D | F | G | C | A | T | 394 | + | 71.9 |
| At1g03740.1\_ | 309 | F | T | E | P | Q | V | K | C | Y | M | R | Q | L | L | S | G | L | E | H | C | H | . | . | . | S | R | G | V | L | H | R | D | I | K | G | S | N | L | L | I | D | S | K | G | . | . | . | . | . | . | . | . | V | L | K | I | A | D | F | G | L | A | T | 360 | + | 98.9 |
| At1g03920.1\_ | 232 | L | S | E | D | E | A | K | F | Y | I | A | E | S | V | L | A | I | E | S | I | H | . | . | . | N | R | N | Y | I | H | R | D | I | K | P | D | N | L | L | L | D | R | Y | G | . | . | . | . | . | . | . | . | H | L | R | L | S | D | F | G | L | C | K | 283 | + | 77.0 |
| At1g03930.1\_ | 100 | L | S | L | K | T | V | L | M | L | A | D | Q | L | I | N | R | V | E | F | M | H | . | . | . | T | R | G | F | L | H | R | D | I | K | P | D | N | F | L | M | G | L | G | R | K | A | N | . | . | . | . | . | Q | V | Y | I | I | D | F | G | L | G | K | 154 | + | 89.5 |
| At1g04210.1\_ | 899 | V | P | M | D | L | A | L | S | I | A | R | D | I | S | G | A | L | M | E | L | H | . | . | . | S | K | D | I | I | H | R | D | I | K | S | E | N | V | L | I | D | L | D | N | Q | S | A | N | G | E | P | . | I | V | K | L | C | D | F | D | R | A | V | 957 | + | 55.1 |
| At1g04440.1\_ | 100 | F | S | L | K | T | V | L | M | L | A | D | Q | M | I | N | R | V | E | Y | M | H | . | . | . | V | K | G | F | L | H | R | D | I | K | P | D | N | F | L | M | G | L | G | R | K | A | N | . | . | . | . | . | Q | V | Y | I | I | D | Y | G | L | A | K | 154 | + | 88.7 |
| At1g04700.1\_ | 867 | L | D | R | R | K | K | L | M | I | T | L | D | S | A | F | G | M | E | Y | L | H | . | . | . | M | K | N | I | V | H | F | D | L | K | C | D | N | L | L | V | N | L | R | D | P | Q | R | P | . | . | . | . | I | C | K | V | G | D | F | G | L | S | R | 922 | + | 69.8 |
| At1g05100.1\_ | 102 | I | D | E | A | R | V | V | K | Y | T | R | Q | I | L | L | G | L | E | Y | I | H | N | . | . | S | K | G | I | A | H | C | D | I | K | G | S | N | V | L | V | G | E | N | G | . | . | . | . | . | . | . | . | E | A | K | I | A | D | F | G | C | A | K | 154 | + | 84.4 |
| At1g05700.1\_ | 653 | L | S | W | R | Q | R | L | Q | I | A | L | D | A | A | Q | G | L | E | Y | L | H | C | G | C | K | P | P | I | V | H | R | D | V | K | T | S | N | I | L | L | N | E | K | N | . | . | . | . | . | . | . | . | R | A | K | L | A | D | F | G | L | S | R | 707 | + | 81.7 |
| At1g06390.1\_ | 166 | M | P | I | F | Y | V | K | L | Y | T | Y | Q | I | F | R | G | L | A | Y | I | H | T | . | . | V | P | G | V | C | H | R | D | V | K | P | Q | N | L | L | V | D | P | L | T | H | . | . | . | . | . | . | . | Q | V | K | L | C | D | F | G | S | A | K | 219 | + | 106. |
| At1g06390.2\_ | 166 | M | P | I | F | Y | V | K | L | Y | T | Y | Q | I | F | R | G | L | A | Y | I | H | T | . | . | V | P | G | V | C | H | R | D | V | K | P | Q | N | L | L | V | D | P | L | T | H | . | . | . | . | . | . | . | Q | V | K | L | C | D | F | G | S | A | K | 219 | + | 106. |

|  |  |
| --- | --- |
| Score:  **1289.52** |  |
| logo with ssc |  |

 **against sequence databases using GLAM2SCAN.**

  


  
*Regular Expression for Motif:* 
 **L[KST][EW]DE[ARV][KRL]R[YI][AT]LQ[IL][AI]N[AG][LV][AE]Y[ICL]H.{0,3}S[PKR]G[IV][LVY]HR?D[ILV]KP[SED]N[IL]L[LV]D[AL].{1,10}[QV][ALV]K[ILV]SDFGL[AS]K** 
  

## Replicates:

Check that at least some of these are similar to the best motif found.  
If not, there may well be an even better motif.  
Click here to
with double the "maximum number of iterations without improvement" to find it.

|  |  |
| --- | --- |
| Score:  **1252.93** |  |
| logo with ssc |  |

 **against sequence databases using GLAM2SCAN.**

  


  
*Regular Expression for Motif:* 
 **[KRL]R[YI][AT]LQ[IL][AI]N[AG][LV][AE]Y[ICL]H.{0,3}S[PKR]G[IV][LVY]HR?D[ILV]KP[SED]N[IL]L[LV]DP?[ARL]?NG.{0,7}[QV][ALV]K[ILV]SDFGL[AS]KF[LS]R** 
  

|  |  |
| --- | --- |
| Score:  **1240.60** |  |
| logo with ssc |  |

 **against sequence databases using GLAM2SCAN.**

  


  
*Regular Expression for Motif:* 
 **[KRL]R[YI][AT]LQ[IL][AI]N[AG][LV][AE]Y[ICL]H[TN]?S[KR]?G.{0,4}[IV][LVY]HR?D[ILV]KP[SED]N[IL]L[LV]DA?NG[NV][QAL].{0,8}K[ILV]SDFGL[AS]KF[LS]R** 
  

|  |  |
| --- | --- |
| Score:  **1234.97** |  |
| logo with ssc |  |

 **against sequence databases using GLAM2SCAN.**

  


  
*Regular Expression for Motif:* 
 **[YI][AT]LQ[IL][AI]N[AG][LV][AE]Y[ICL]HS.{0,3}[PKR]G[IV][LVY]HR?D[ILV]KP[SED]N[IL]L[LV]D[AL].{0,9}G[QV][ALV]K[ILV]SDFGL[AS]KF[LS]R.{0,19}[STG][HEL][LPS][HSV]** 
  

|  |  |
| --- | --- |
| Score:  **913.469** |  |
| logo with ssc |  |

 **against sequence databases using GLAM2SCAN.**

  


  
*Regular Expression for Motif:* 
 **[LT]S[NTY]V[CT].{0,1}G?TPRY[MRV]?APE?[LV]L?[LF]?N[ACD]?[KT][GH]Y.{0,4}[TG]A[AE].{0,7}[SV]D?[IV][YW]S[FL]G[CV][ILV]L[FM]E[IL][IML][AT]G[KSY][LN]?P[FL]F?[DP][EG]S[NR]** 
  

|  |  |
| --- | --- |
| Score:  **699.274** |  |
| logo with ssc |  |

 **against sequence databases using GLAM2SCAN.**

  


  
*Regular Expression for Motif:* 
 **[VY]VG[PT][PF][DR]Y[MR]?APELL.[GKT]G.{0,8}I?C?[EK]Y[GST][AE][EKS][CSV]DP?[IW][YWF]S[FL]G[AC][LV]LMI?E[IL][IL][LT]G[KS][NP][PL][FI]?[PT]G[LS][SR]** 
  

|  |  |
| --- | --- |
| Score:  **673.886** |  |
| logo with ssc |  |

 **against sequence databases using GLAM2SCAN.**

  


  
*Regular Expression for Motif:* 
 **[GKT]GYE?Y[STG][AE][AEK][SV]D[IV][YW]S[FL]G[CV][ILV]L[FM]E[IL][IML][AT]G[KSY][LN]?P[FL][DF][EP][GLS]RL[MQ][TV]LQ** 
  

|  |  |
| --- | --- |
| Score:  **458.308** |  |
| logo with ssc |  |

 **against sequence databases using GLAM2SCAN.**

  


  
*Regular Expression for Motif:* 
 **RHQ[DT]V?[KRE][EQ][FL][AK][RV].{0,1}E[ILV]E[LV][ML][GKR]R[LV]?[DKR].{0,1}HPN[ILV][IV]?E[IYL][ILY]G[VY]F.[ES]** 
  

|  |  |
| --- | --- |
| Score:  **299.297** |  |
| logo with ssc |  |

 **against sequence databases using GLAM2SCAN.**

  


  
*Regular Expression for Motif:* 
 **L[IL][GKS]Y[CFT][DS]?[ERV]E[EGK].[YF]?[LNR].{0,2}[HY][KS]?[ML]V[EMY][ED][YLF][MV][DPR].{0,2}[EGS][TD][ML]** 
  

|  |  |
| --- | --- |
| Score:  **214.607** |  |
| logo with ssc |  |

 **against sequence databases using GLAM2SCAN.**

  


  
*Regular Expression for Motif:* 
 **DV[DS]DE[DS]I?T[IEG]?NH[SCV][DF][SF]?[AS]TFS[LN]?S?[EK]E[CSV]?[RL]V[TL][AEN]K**
